# Surgical Decompression of Painful Diabetic Peripheral Neuropathy: The Role of Pain Distribution

**DOI:** 10.1371/journal.pone.0109827

**Published:** 2014-10-07

**Authors:** Chenlong Liao, Wenchuan Zhang, Min Yang, Qiufeng Ma, Guowei Li, Wenxiang Zhong

**Affiliations:** Department of Neurosurgery, XinHua Hospital, affiliated to Shanghai JiaoTong University School of Medicine, Shanghai, P. R. China; University of Michigan Medical School, United States of America

## Abstract

**Objective:**

To investigate the effect of surgical decompression on painful diabetic peripheral neuropathy (DPN) patients and discuss the role which pain distribution and characterization play in the management of painful DPN as well as the underlying mechanism involved.

**Methods:**

A total of 306 patients with painful diabetic lower-extremity neuropathy were treated with Dellon surgical nerve decompression in our department. Clinical evaluation including Visual analogue scale (VAS), Brief Pain Inventory Short Form for diabetic peripheral neuropathy (BPI-DPN) questionnaire, two-point discrimination (2-PD), nerve conduction velocity (NCV) and high-resolution ultrasonography (cross-sectional area, CSA) were performed in all cases preoperatively, and at 6 month intervals for 2 years post-decompression. The patients who underwent surgery were retrospectively assigned into two subgroups (focal and diffuse pain) according to the distribution of the diabetic neuropathic pain. The control group included 92 painful DPN patients without surgery.

**Results:**

The levels of VAS, scores in BPI-DPN, 2-PD, NCV results and CSA were all improved in surgical group when compared to the control group (*P*<0.05). More improvement of VAS, scores in BPI-DPN and CSA was observed in focal pain group than that in diffuse group (*P*<0.05).

**Conclusions:**

Efficacy of decompression of multiple lower-extremity peripheral nerves in patients with painful diabetic neuropathy was confirmed in this study. While both focal and diffuse group could benefit from surgical decompression, pain relief and morphological restoration could be better achieved in focal group.

## Introduction

There are over 250 million people in the world with type 1 and 2 diabetes mellitus. [1] Neuropathy is one of the most common complications of diabetes mellitus and leads to increasingly high morbidity and mortality, resulting in a huge economic burden for diabetes care. [2] Diabetic neuropathy is a heterogeneous condition containing symmetrical neuropathies and focal neuropathies, [3] presenting diverse clinical manifestations. Of all the neuropathies in diabetes, chronic diabetic peripheral neuropathy (DPN) is the commonest. [4] Of all the symptoms in DPN, pain is the most distressing and is the main factor that prompts the patients to seek medical advice. [5] One-third of diabetic patients have symptoms of neuropathic pain according to a recent community-based study [6] and up to 15–20% of patients with DPN may experience painful symptoms. [7] Therefore, a high proportion of patients are suffering from neuropathic pain as well as the relative depression, anxiety and sleep deprivation.

The management of neuropathic pain in diabetes still remains challenging mainly due to its various clinical features, wide spectrum severity and different distribution involved. Descriptions of pain can be burning, prickling, lancinating, shooting, cramping, aching, and also contact hypersensitivity (allodynia) and “dead feeling” (numbness) in their legs. [5] The severity may range from mild symptoms in one toe or two to continuous painful symptoms involving both legs and may even extend to the upper limbs. The extent involved may be focal or diffuse. One additional factor that contributes to the treatment dilemma of neuropathic pain is the varied response to the currently different treatments. The diverse manifestations of neuropathic pain in diabetes and various responses to current interventions imply that a number of mechanisms could contribute. Therefore, the management of painful DPN may not be one single intervention and a series of factors should be taken into consideration, one of which, as Vinik, A. and his colleagues put it in one guideline, [8] may be the distribution of pain. According to our clinical experience with management of painful diabetic neuropathy, features and severity of pain may change during the course of diabetic neuropathy while the distribution of pain is relatively invariable, which may be of some value for patient selection for surgical decompression. Thus we carry out this retrospective study to investigate the effects of surgical decompression on the outcome of painful diabetic patients and discuss the role which pain distribution plays in the management of painful diabetic neuropathy as well as the underlying mechanism involved.

Aside from traditional management including glucose control, lifestyle modification and pharmacological treatment, surgical decompression is recommended for pain relief in the recent reports [9]–[15] based on the “double crush” hypothesis. [16] Clinical observations revealed that many of the symptoms of diabetic neuropathy, including pain, are similar to those of chronic nerve compression, suggesting that entrapment of nerves may happen in the patients with diabetic neuropathy. Allowing for the currently traditional treatment dilemma on pain relief in patients with diabetic neuropathy, surgical decompression targeting superimposed compression, as an newly emerging promising approach, should be impartially taken into account.

## Methods

This study is approved by the Xinhua Hospital Ethics Review Board and the forms of consent were obtained from all patients involved in this study.

### Patients

A consecutive series of 306 patients (108 males and 198 females) with painful diabetic lower-extremity neuropathy, who underwent Dellon surgical nerve decompression [17] in our department from January 2008 to December 2011 was collected in this study. The control group included 92 painful DPN patients (38 males and 54 females) without surgery. The control group was mainly made up of the outpatients who refused hospitalization for surgical intervention owing to various personal reasons, most of which included fear of surgery and economic issues. Patients in the control group were all qualified for the inclusion criteria described below. According to the Wagner classification, 77 patients in surgical group (25.2%) and 26 patients in control group (28.3%) were rated as class 1 (surface ulcers and no clinical infection) before surgery. All patients had a history of type II diabetes mellitus according to 1999 WHO diagnostic criteria and showed symptoms of neuropathic pain, with high Toronto clinical scoring system (TCSS) scores. According to the definition proposed by the International Association for the Study of Pain, neuropathic pain in diabetes is “pain arising as a direct consequence of abnormalities in the somatosensory system in people with diabetes”. [18] After reviewed the histories, we retrospectively defined the focal pain (the pain mainly confined to one to three scattered areas of the legs, dorsum of feet, the heels, the toes, or the plantar aspect of feet) and the diffuse pain (the pain is so dispersed along the affected extremities that exactly position can not be localized) and then divide the surgical group into two subgroups according to this definition. The focal pain was observed in 145 patients and the diffuse pain in the remaining 161 patients. The baseline characteristics of patients are displayed in Table 1.

**Table 1:** Baseline of characteristics of patients.

	Control(n = 92)	Focal(n = 145)	Diffuse(n = 161)	*P*(a, b)*
Patient characteristics				
Median age(yr) (%)	57±13.62(36–86)	60±11.59(34–86)	58±11.36(36–85)	0.08, 0.93
Age in years, n (%)				
<40	9(9.7)	7(4.8)	11(6.8)	
40–49	21(22.8)	26(17.9)	31(19.3)	
50–59	25(27.1)	36(24.8)	39(24.2)	
60–69	22(23.9)	62(42.8)	67(41.6)	
>70	15(16.3)	14(9.7)	13(8.1)	
Sex, n (%)				0.29, 0.84
Male	38(41.3)	52(35.9)	56(34.8)	
Female	54(58.7)	93(64.1)	105(65.2)	
Course of DM(yr)	7.2±1.62	7.7±2.72	8.1±1.85	0.83, 0.78
Course of pain(mo)	47±5.38	43±5.61	57±6.32	0.80, 0.74

All variables were expressed in mean ± SD.

DM = Diabetes Mellitus.

*a: control group vs. focal & diffuse (surgical group), b: focal group vs. diffuse group.

Inclusion criteria include (1) history of pain in the distribution of the posterior tibial (medial and lateral plantar surface), common and superficial peroneal (lateral calf and dorsum of the foot), or deep peroneal nerves (dorsum of the foot, first web space), (2) positive Tinel sign presented at a known site of nerve entrapment including the fibular tunnel at the lateral side of the knee, for the common peroneal nerve, the tarsal tunnel at the ankle for the tibial nerve, and the junction between the first and second metatarsals and the cuneiforms for the deep peroneal nerve, (3) Decreased two-point discrimination: the big toe pulp two-point discrimination was greater than 9 mm [19].

Exclusion criteria include (1) presence of defined risk factors such as alcohol, nutrition, uremia and peripheral vascular disease, as demonstrated by absence of a palpable pulse, (2) previous history of cervical and lumbar spondylosis, (3) radiculopathy, (4) pedal edema.

### Surgical Technique

The decompression surgery adopting Dellon triple procedures [17] were performed by the same senior surgeon (Zhang WC), with the use of microscope under continuous epidural anesthesia.

A 3 cm-long incision was made below the fibular head and the skin, superficial and deep fascia was cut. After excision of fascia above the common peroneal nerve and the peroneus longus muscle tendon, the common peroneal nerve trunk was exposed and decompressed.

A 6 cm-long curved incision was made along the medial malleolus and the skin, superficial and deep fascia was cut. After excision of the flexor retinaculum, the posterior tibial artery and veins and the tibal nerve were indentified and decompressed. Following this, the abductor halluces brevis was cut and spread to expose and divide the roof of the medial and of the lateral plantar tunnels. The medial calcaneal tunnel was then identified and decompressed. Epineurium decompression was performed if there was evidence of epineurial thickening.

A 2 cm-long incision was made longitudinally between the first and second metatarsal heads and the skin, superficial and deep fascia were cut to expose the tendon of the extensor hallucis brevis muscle. This tendon was then excised to decompress the deep peroneal nerve.

During the perioperative period, blood glucose was controlled at 6.2–8.0 mmol/L. After the patients were discharged, they were required to monitor their fasting plasma glucose every week and control their blood glucose levels to <8.0 mmol/L.

### Clinical evaluation

Careful history taking and peripheral neurological/vascular examination were completed in all patients at admission. Aside from pain as a chief complaint, some of them simultaneously presented with other sensory symptoms including numbness, tingling and so forth. Patients were additionally inquired about their functional status, normal activities, ability to work, walking distance, family history of diabetes and the use of medications.

Assessment of pain including visual analogue scale (VAS) and Brief Pain Inventory Short Form for diabetic peripheral neuropathy (BPI-DPN), of sensation including plantar big toe and small toe two-point discrimination (2-PD), of morphological changes of nerves employing high-resolution ultrasound, of electrophysiological changes performing nerve conduction velocity (NCV) on admission prior to surgery and then at 6 months intervals for two years after surgery. The follow-up was conducted mainly on an outpatient basis.

Pre and post operative evaluations were performed by two clinical residents (in charge of history taking and peripheral neurological/vascular examination), one sonographer (undertaking high-resolution ultrasound of peripheral nerves) and one technician of electrophysiology (performing the NCV tests).

The visual analogue scale (VAS) [20], which is one of the oldest and best validated measurement (0 = no pain to 10 = worst possible pain), was used to assess the severity of diabetic neuropathic pain. All patients were asked to complete Brief Pain Inventory Short Form for diabetic peripheral neuropathy (BPI-DPN) to assess pain severity and pain interference with daily functioning. The BPI-DPN includes the four-item pain Severity scale (Worst Pain, Least Pain, Average Pain, and Pain Now) and the seven-item pain Interference scale (General Activity, Mood, Walking Ability, Normal Work, Relations with Others, Sleep, Enjoyment of Life). Each items of BPI uses a 0 to 10 numeric rating scale anchored at “no pain,” and 10 for “pain as bad as you can imagine” for Severity, and “does not interfere” to “completely interferes” for Interference [21].

Neurosensory examination mainly included percussion over a distribution of the affected peripheral nerve (Tinel sign) and two-point discrimination (2-PD) using the Disk-Criminator at the big and small toes for medial and lateral plantar nerves, respectively. A positive Tinel sign was taken to be a positive response that indicated either a tingling or radiating electriclike perception either into the heel, the arch, or the toes (the most common responses), or proximally up the inside of the ankle (the least common response) [22].

Nerve conduction velocity was detected with the employment of a Denmark Medtronic EMG (DK 1 2740). The testing was performed under a quiet indoor environment with a room temperature of 25°C and skin temperature of 30°C, using surface electrodes for stimulation and recording. Bilateral motor NCVs of tibial nerve and sensory NCVs of the common peroneal nerve and superficial peroneal nerve were recorded in all patients.

High-resolution ultrasound of peripheral nerves was performed employing a Sequoia 512 ultrasound device (Siemens) with a 8–14 MHz transducer. Multislice, longitudinal, and transverse scans were made through the common peroneal nerve and the posterior tibial nerve to observe the continuity and echogeneity of the nerves. The anteroposterior diameter (Da) and transverse diameter (Dt) of the nerves were measured at the cross-section of 1.5 cm below the distal tip of fibular head (common peroneal nerve) and distal tip of medial malleolus (posterior tibial nerve). And then cross-sectional area (CSA) was calculated by the formula of S = π(Da*Dt/4) [23].

### Statistical Methods

Analysis was performed separately for patients comparing the change from preoperative levels to that of last visit (BPI-DPN, 2-PD, NCV, CSA). Data were expressed as the mean ± standard deviation. Pearson’s χ^2^ test and student's t-test were used with the employment of SPSS 18.0. A *P* value <0.05 was considered statistically significant.

## Results

Twenty-five patients in surgical groups and six patients in control group were lost during the follow-up. The differences of age, sex, course of diabetes and pain symptom between the two groups were not statistically significant (*P*>0.05). The average courses of diabetes mellitus were longer than the average courses of pain symptom in all groups. In some individuals, however, the onset of pain symptom came before the diagnosis of diabetes, some may even lead to the diagnosis of diabetes, in which situation other potential diseases had been excluded before the diagnosis of diabetic neuropathy. There is no statistically significant difference between surgical and control groups and, also no statistically significant difference among the three groups (focal pain group, diffuse pain group and control group) after dividing the surgical group into two subgroups regarding to the initial VAS level, 2-PD, scores in BPI-DPN, NCV and CSA (*P*>0.05).

### Visual Analogue Scales

**Figure 1:**
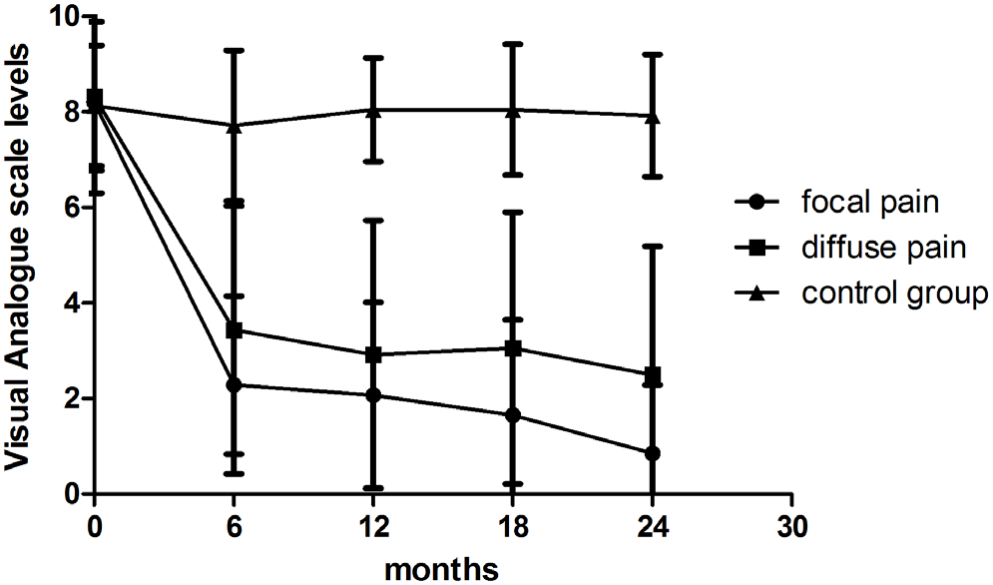
The figure displays the records of VAS levels at different time points. The VAS scores in both surgical groups showed notable decline with the time course while no changes occurred in control group during the follow-up. The overall postoperative VAS levels of focal pain group were much lower than that of diffuse pain group (*P*<0.01).

The VAS levels in different time points were recorded and comparison was made among the three groups to assess the outcome of surgery. (Figure 1) In focal pain group, the average score of VAS was 8.20 on admission and at 6 months after surgery decreased to 2.28 (*P*<0.01). The average pain level remained between 2 and 1 for the remainder of the study. The mean VAS pain level of patients with diffuse pain was 8.32 and decreased to 3.43 (*P*<0.01) 6 months after surgery, higher than that of patients with focal pain (*P*<0.05). The mean pain level of diffuse pain group remained between 4 and 2 with fluctuation for the remainder of the study. The average VAS of patients in control group was 8.03 at first visit and remained around 8.0 during the two-year follow-up (*P*>0.05). There was a statistically significant difference as to VAS level between surgical and control groups after surgery (*P*<0.01). The results show pain relief can be achieved through surgical decompression, especially in the patients with focal pain.

### Two-point discrimination

**Figure 2:**
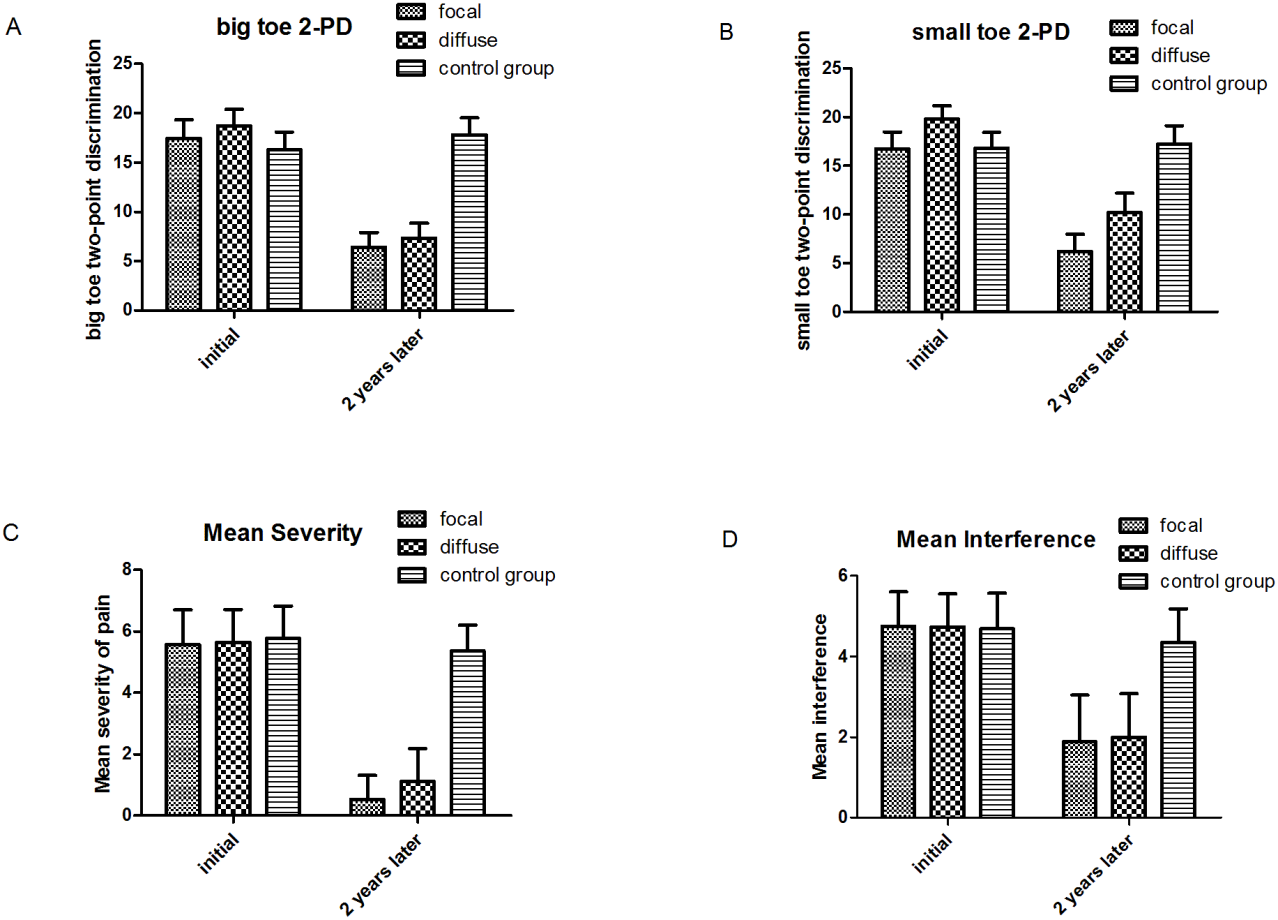
Graphs illustrate results of two-point discrimination and BPI-DPN in all groups. A, B, both surgical groups show marked decline in plantar big toe and small toe 2-PD while no apparent improvement can be observed in the control group; C, both surgical groups exhibit significant alleviation in pain severity through surgery while the control group remained at nearly the same level of mean severity of pain; D, decrease in the mean activity interference was displayed in both surgical groups and almost no changes existed in the control group.

In focal pain group, the postoperative (two years later) average big toe 2-PD (6.43±1.46 mm) and small toe 2-PD (6.24±1.72 mm) showed a statistically significant improvement (*P*<0.05) comparing to those before surgery (17.4±1.86 mm, 16.7±1.71 mm). Analogously, markedly improvement can also be observed when comparison was made between preoperative (18.7±1.68 mm, 19±1.34 mm) and postoperative (7.34±1.54 mm, 10.24±1.94 mm) big toe and small toe 2-PD in diffuse pain group (*P*<0.05). There is no statistically significant difference between two groups with regard to both big toe 2-PD (*P = *0.73) and small 2-PD (*P = *0.77) after decompression. In control group, no statistically significant difference could be seen between preoperative and postoperative 2-PD of big toe (pre: 16.3±1.77 mm, post: 17.8±1.72 mm, *P*>0.05) and small toe (pre: 16.3±1.77 mm, post: 16.8±1.57 mm, *P*>0.05). A statistically significant improvement in both small toe and big toe 2-PD could be observed between surgical and control groups (*P*<0.05) (Figure 2 A, B).

### BPI-DPN questionnaire

The scores of all items in BPI-DPN were recorded, and the “Mean severity” as well as the “Mean interference” was compared in this study. (Figure 2 C, D) In the focal pain group, the preoperative scores of “Mean severity” (5.56±1.14) decreased greatly after two years (0.52±0.78) (*P*<0.05). There is also an observable decline (5.64±1.07, 1.12±1.01) in “Mean severity” two years after surgery in the diffuse pain group (*P*<0.05). Initial “Mean interference” of focal and diffuse pain group is 4.75±0.86 and 4.73±0.82, which decreased to 1.88±1.15 (*P*<0.05) and 2.00±1.07 (*P*<0.05) respectively. Statistically significant difference existed between the two groups regarding both postoperative “Mean severity” (*P*<0.05) and “Mean interference” (*P*<0.05). As respect to “Mean severity” (preoperative: 5.77±1.05, postoperative: 5.37±0.83) and “Mean interference” (preoperative: 4.69±0.88, postoperative: 4.36±0.83), there was almost no changes in control group.

### Nerve conduction velocity (NCV) study

Two years after surgery, NCVs of all the patients increased significantly compared to that before surgery (*P*<0.05) while NCVs of patients in control group remained almost unchanged. Statistically significant difference could be observed between the surgical and control groups (*P*<0.05) while no statistically significant difference existed between the focal and diffuse pain group (*P = *0.84) two years after surgery (Table 2).

**Table 2:** Pre and post operative NCVs of DPN patients (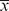
±S).

	Preoperative NCV(ms)			Postoperative NCV(ms)		
	Control Group	Focal Group	Diffuse Group	Control Group	Focal Group	Diffuse Group
Posterior tibial nerve	36.7±3.4	33.5±3.1	35.5±3.6	31.6±3.5	41.8±2.6	42.0±2.8
Commonperoneal nerve	33.8±2.8	37.8±3.4	36.8±1.9	28.6±3.3	42.5±2.4	42.2±2.9
Superficialperoneal nerve	38.6±3.7	35.8±2.5	37.6±2.9	34.4±3.6	42.2±3.1	43.6±2.3
Sural nerve	37.4±2.8	37.8±3.4	37.7±3.9	37.0±2.5	43.6±2.7	43.2±3.6

All variables were expressed in mean ± SD.

NCV: nerve conduction velocity.

Nerve decompression increases NCV in operated cases by 15–20%.

### High-resolution ultrasonography

**Figure 3:**
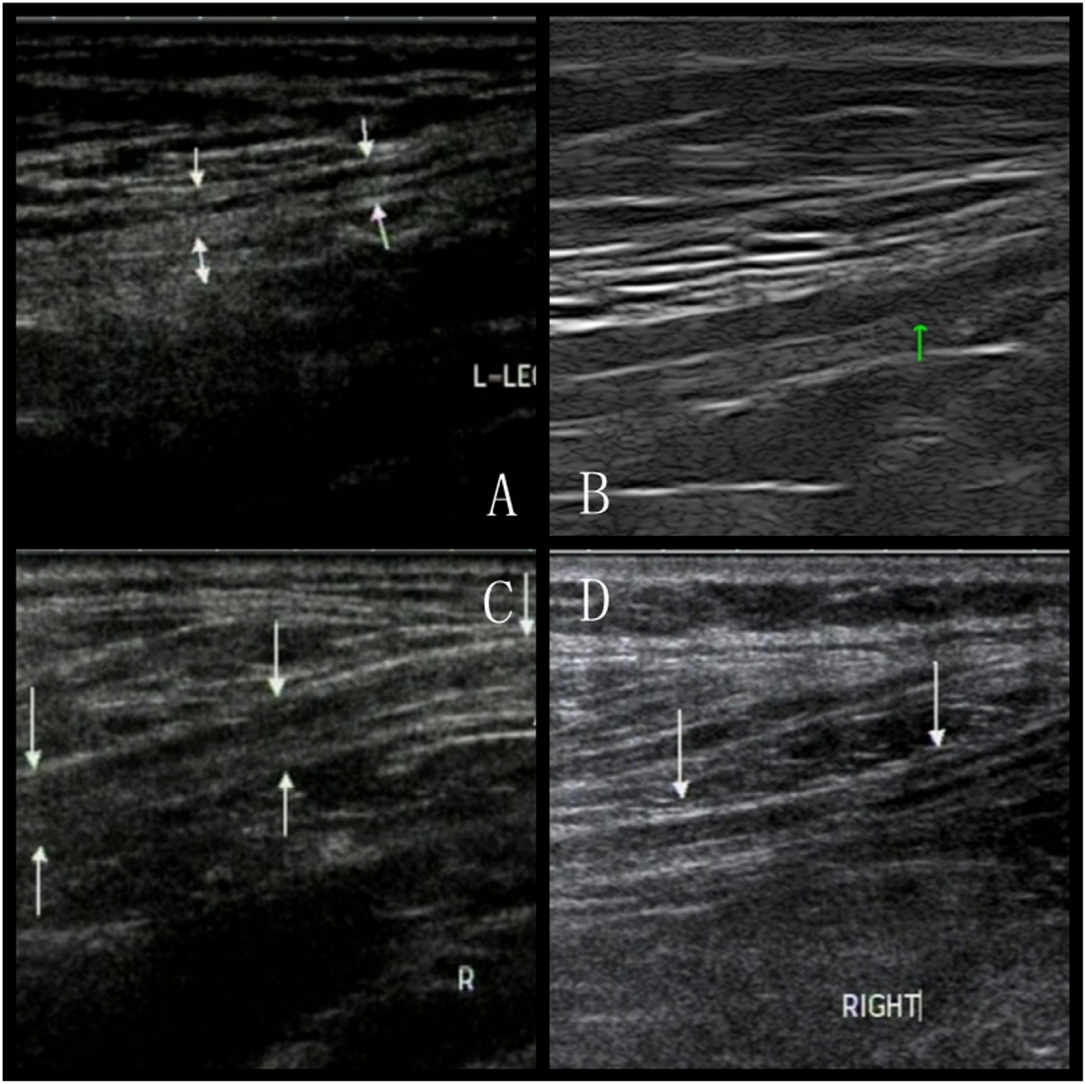
Longitudinal sonograms showing difference of morphological changes after surgical decompression between two patients with focal and diffuse painful DPN. A and B represent the preoperative (Da: 4.4 mm, Dt: 7.6 mm) and postoperative (Da: 2.9 mm, Dt: 6.3 mm) sonograms of the common peroneal nerve at the level of knee joint of patient with focal painful DPN. C and D represent the preoperative (Da: 4.2 mm, Dt: 7.0 mm) and postoperative (Da: 3.7 mm, Dt: 6.8 mm) sonograms of the common peroneal nerve at the level of knee joint of patient with diffuse painful DPN.

In all groups, fusiform swelling of the nerves, intraneural hypoecho and, disappearance of intraneural parallel and linear structure were observed by high-resolution ultrasonography. Two years after surgery, the restoration of nerve structure can be seen in both focal and diffuse pain group. More decrease of CSA was seen in patients with focal pain (*P*<0.05). (Figure 3) The CSA of patients in control group remained larger than that of both focal (*P*<0.05) and diffuse (*P*<0.05) pain group after surgery (Table 3).

**Table 3:** Pre and post operative CSA of DPN patients (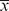
±s).

	Preoperative CSA			Postoperative CSA		
	Control Group	Focal Group	Diffuse Group	Control Group	Focal Group	Diffuse Group
Tibial nerveCSA(mm^2^)	24.4±4.2	25.3±3.5	25.1±4.1	25.1±3.6	16.9±3.2	19.7±3.8
Common peronealnerve CSA(mm^2^)	21.6±3.7	20.8±3.5	21.2±4.0	21.9±3.2	14.6±2.8	15.9±3.5

All variables were expressed in mean ± SD.

CSA: cross-sectional area.

All patients underwent surgical decompression recovered well except that three patients suffered from surgical complications. Wound dehiscence in two patients healed within 2 months after strict blood glucose control, antibiotics administration and daily dressing change. One patient with extensive lower-leg subcutaneous hemorrhage was treated by surgical incision and drainage. In the control group, ten patients developed superficial ulcers in the lower-limb and two underwent surgical amputation during this period. None of the operated patients had developed new ulcer, ulcer progression or amputation in two-year follow-up.

## Discussion

Diabetic neuropathy has traditionally been considered as an irreversible condition and the treatments are mostly symptomatic aiming to prevent the development of complications rather than to target the underlying pathologic mechanisms. [24] However, the proposal of “double crush” hypothesis contributed by the combination of increase endoneurial water content [25], [26] and consequent slow axoplasmic flow [27]–[29] offers hope to people with diabetic neuropathy and superimposed nerve compression since neurolysis of these entrapped nerves may give symptomatic relief and possess potential for reversibility. As such, over the past two decades, surgical decompression of peripheral nerves has become an increasingly popular method for treating patients suffering from painful diabetic neuropathy.

Painful DPN can be divided into two varieties: acute and chronic painful DPN. Here we mainly focus on chronic painful DPN due to its highly prevalence and the facts that acute painful DPN is relatively rare and the acute symptoms may resolve within a year. [30], [31] According to our clinical experience with management of painful diabetic neuropathy, pain is a heterogenous sensation and patients may describe their symptoms in markedly different ways. Features and severity of the pain may change with the development of diabetic neuropathy while the extent of pain is relatively invariable, which may be of somewhat value. We observed that some patients with painful DPN localized their pain mainly to the scattered areas of the legs, dorsum of feet, the heels, the toes, or the plantar aspect of feet while some others complained the pain was so dispersed that it even involved the whole lower limbs and the exact position could not be localized. Inspired by one previous guideline [8], we retrospectively defined the former as focal pain and the later as diffuse pain, and divided a series of 306 painful diabetic patients who underwent decompression surgery into two subgroups in the light of this definition. In this study we firstly compared the surgical result between surgical group and control group and, confirmed the efficacy of these decompression surgeries. Then we compared the surgical result of pain relief in patients of surgical group, revealing different responses of patients with focal and diffuse pain to the surgical decompression: people with focal painful DPN show a greater potential to achieve pain relief. Morphologically, in addition, better ultrasound restoration could be observed in focal pain group than diffuse pain group.

The answer to the question of “what is the pathophysiology behind the distinct reaction to the decompression between patients with focal or diffuse painful DPN” should be started with the mechanisms of neuropathic pain in diabetes. Although the exact pathophysiological mechanisms of neuropathic pain in diabetes remain enigmatic, several mechanisms including peripheral and central mechanisms have been postulated based on the result of experiments in animal model of neuropathic pain. [32]–[36] It is suggested that all levels of the nervous system, from peripheral nerve to the brain, were affected by the diabetic neuropathy. [37] Both metabolic and mechanical (compressive) mechanism may be the source of the painful symptom.

Diabetes causes deposition of collagen in the small arteries that supply the peripheral nerves, hypothetically resulting in a length-dependent sensorimotor neuropathy. Increased aldose reductase activity in diabetes converts glucose to sorbitol, which is hydrophilic and draws water into the nerve, rendering the peripheral nerve susceptible to mechanical compression and decreasing the slow axoplasmic transport in the diabetic nerve. As a consequence, proteins for structural repairs could not be transported to the impaired site within the diabetic nerve. [38] Injured peripheral nerve fibers give rise to the intense and prolonged input of ectopic activity to the central nervous system. After that, neurons in the spinal and the brain may change their response characteristics and exhibit signs of hyperexcitability in a fashion, mimicking that presented after peripheral nerve injury [39].

Given all that above, in the early stage of some cases, nerve thickening is suggested to play a fundamental role in the pathophysiology of painful diabetic neuropathy, with a manifestation of focal pain. As such, it is conceivable that entrapment of affected nerves at sites of anatomic narrowness may occur, which could be treated through surgical decompression. As the compression continues, however, neuroplastic changes in central nervous system happen after reorganization of structures participating in the processing of noxious information, which may present with diffuse pain in certain patients. The management is rather complicated. Aside from the current medical therapies including tricyclic antidepressants, the serotonin and noradrenaline re-uptake inhibitor and anti-convulsants, and so forth, an unmet need still exists for pharmacological agents targeting the underlying mechanisms due to the ineffectiveness and the side effects of the available drugs. In this study, the severity of pain, two-point discrimination, NCV results and morphological changes (SCA) of compressed nerve were all improved in surgical group when compared to the control group. Furthermore, there is no significant difference between the focal and diffuse pain groups regarding the results of two-point discrimination and NCV testing, which was increased by 15–20% after nerve decompression. (Table 2) Therefore, surgical decompression is also encouraged among the patients with diffuse painful DPN when the Tinel sign is positive, which indicates the existence of entrapment and regeneration of axons [40].

The limitations in our study include: (1) although being queried on admission, non-surgical treatment such as pharmacotherapy of neuropathic pain was not precisely measured among all the patients due to the retrospective nature of this study. For this reason, preoperative evaluation including pain levels was performed with both VAS and BPI-DPN and no significant difference was observed. (2) The specific definition of the focal and diffuse pain. Although extent of pain in most patients is relatively invariable, parts of patients would experience both focal and diffuse pain during the course of painful DPN. The phenotype of pain was decided on admission prior to surgery. Further prospective, randomized and controlled trial with blinded study executers and observers is needed to verify the role of peripheral nerve decompression in patients with painful DPN and further elucidate the role which pain distribution and characterization play in managing painful diabetic neuropathy as well as the underlying mechanism involved.

Allowing for the mixed results reported and the continuing controversy among researchers as to the effectiveness of nerve decompression surgery on DPN, [9], [10], [12], [13], [15], [17], [41]–[46] patient selection for surgery becomes increasingly critical through the long march to definitely delineate and confirm the place for nerve decompression. Distribution of pain indeed plays a practical role in predicting the response of painful DPN to the surgery according to the result of this study.

## Conclusions

The results of this study continue to support the efficacy of decompression of multiple lower-extremity peripheral nerves in patients with painful diabetic neuropathy who presented with a positive Tinel sign. Favorable outcomes could be achieved in treating focal painful DPN through surgical decompression, which would also be helpful and should not be abandoned in diffuse painful DPN in the presence of Tinel sign.

## References

[1] Shaw JE, Sicree RA, Zimmet PZ (2010) Global estimates of the prevalence of diabetes for 2010 and 2030. Diabetes Res Clin Pract 87: 4–14. 19896746 10.1016/j.diabres.2009.10.007

[2] Vinik AI, Park TS, Stansberry KB, Pittenger GL (2000) Diabetic neuropathies. Diabetologia 43: 957–973. 10990072 10.1007/s001250051477

[3] Thomas PK (1973) Metabolic neuropathy. J R Coll Physicians Lond 7: 154–160. 4348041 PMC5368790

[4] Boulton AJ, Malik RA, Arezzo JC, Sosenko JM (2004) Diabetic somatic neuropathies. Diabetes Care 27: 1458–1486. 15161806 10.2337/diacare.27.6.1458

[5] Quattrini C, Tesfaye S (2003) Understanding the impact of painful diabetic neuropathy. Diabetes Metab Res Rev 19 Suppl 1S2–8. 12577252 10.1002/dmrr.360

[6] Abbott CA, Malik RA, van Ross ER, Kulkarni J, Boulton AJ (2011) Prevalence and characteristics of painful diabetic neuropathy in a large community-based diabetic population in the U.K. Diabetes Care. 34: 2220–2224. 10.2337/dc11-1108 PMC3177727 21852677

[7] Tesfaye S, Boulton AJ, Dickenson AH (2013) Mechanisms and management of diabetic painful distal symmetrical polyneuropathy. Diabetes Care 36: 2456–2465. 23970715 10.2337/dc12-1964 PMC3747929

[8] Vinik AI, Casellini CM (2013) Guidelines in the management of diabetic nerve pain: clinical utility of pregabalin. Diabetes Metab Syndr Obes 6: 57–78. 23467255 10.2147/DMSO.S24825 PMC3587397

[9] Wieman TJ, Patel VG (1995) Treatment of hyperesthetic neuropathic pain in diabetics. Decompression of the tarsal tunnel. Ann Surg 221: 660–664 discussion 664–665. 7794070 10.1097/00000658-199506000-00005 PMC1234690

[10] Aszmann O, Tassler PL, Dellon AL (2004) Changing the natural history of diabetic neuropathy: incidence of ulcer/amputation in the contralateral limb of patients with a unilateral nerve decompression procedure. Ann Plast Surg 53: 517–522. 15602245 10.1097/01.sap.0000143605.60384.4e

[11] Biddinger KR, Amend KJ (2004) The role of surgical decompression for diabetic neuropathy. Foot Ankle Clin 9: 239–254. 15165580 10.1016/j.fcl.2003.12.001

[12] Rader AJ (2005) Surgical decompression in lower-extremity diabetic peripheral neuropathy. J Am Podiatr Med Assoc 95: 446–450. 16166461 10.7547/0950446

[13] Siemionow M, Alghoul M, Molski M, Agaoglu G (2006) Clinical outcome of peripheral nerve decompression in diabetic and nondiabetic peripheral neuropathy. Ann Plast Surg 57: 385–390. 16998329 10.1097/01.sap.0000221979.13847.30

[14] Karagoz H, Yuksel F, Ulkur E, Celikoz B (2008) Early and late results of nerve decompression procedures in diabetic neuropathy: a series from Turkiye. J Reconstr Microsurg 24: 95–101. 18473283 10.1055/s-2008-1064923

[15] Wood WA, Wood MA (2003) Decompression of peripheral nerves for diabetic neuropathy in the lower extremity. J Foot Ankle Surg 42: 268–275. 14566718 10.1016/s1067-2516(03)00313-2

[16] Upton AR, McComas AJ (1973) The double crush in nerve entrapment syndromes. Lancet 2: 359–362. 4124532 10.1016/s0140-6736(73)93196-6

[17] Dellon AL (1992) Treatment of symptomatic diabetic neuropathy by surgical decompression of multiple peripheral nerves. Plast Reconstr Surg 89: 689–697 discussion 698–689. 1546082

[18] Treede RD, Jensen TS, Campbell JN, Cruccu G, Dostrovsky JO, et al (2008) Neuropathic pain: redefinition and a grading system for clinical and research purposes. Neurology 70: 1630–1635. 18003941 10.1212/01.wnl.0000282763.29778.59

[19] Bril V, Perkins BA (2002) Validation of the Toronto Clinical Scoring System for diabetic polyneuropathy. Diabetes Care 25: 2048–2052. 12401755 10.2337/diacare.25.11.2048

[20] Scott J, Huskisson EC (1976) Graphic representation of pain. Pain 2: 175–184. 1026900

[21] Zelman DC, Gore M, Dukes E, Tai KS, Brandenburg N (2005) Validation of a modified version of the Brief Pain Inventory for painful diabetic peripheral neuropathy. J Vasc Nurs 23: 97–104. 16125633 10.1016/j.jvn.2005.06.004

[22] Lee CH, Dellon AL (2004) Prognostic ability of Tinel sign in determining outcome for decompression surgery in diabetic and nondiabetic neuropathy. Ann Plast Surg 53: 523–527. 15602246 10.1097/01.sap.0000141379.55618.87

[23] Zhang W, Zhong W, Yang M, Shi J, Guowei L, et al. (2013) Evaluation of the clinical efficacy of multiple lower-extremity nerve decompression in diabetic peripheral neuropathy. Br J Neurosurg. 10.3109/02688697.2013.798854 23713665

[24] Melenhorst WB, Overgoor ML, Gonera EG, Tellier MA, Houpt P (2009) Nerve decompression surgery as treatment for peripheral diabetic neuropathy: literature overview and awareness among medical professionals. Ann Plast Surg 63: 217–221. 19593109 10.1097/SAP.0b013e31818ba768

[25] Gabbay KH (1973) The sorbitol pathway and the complications of diabetes. N Engl J Med 288: 831–836. 4266466 10.1056/NEJM197304192881609

[26] Jakobsen J (1978) Peripheral nerves in early experimental diabetes: expansion of the endoneurial space as a cause of increased water content. Diabetologia 14: 113–119. 204534 10.1007/BF01263449

[27] Medori R, Autilio-Gambetti L, Monaco S, Gambetti P (1985) Experimental diabetic neuropathy: impairment of slow transport with changes in axon cross-sectional area. Proc Natl Acad Sci U S A 82: 7716–7720. 2415969 10.1073/pnas.82.22.7716 PMC391404

[28] Dahlin LB, Meiri KF, McLean WG, Rydevik B, Sjostrand J (1986) Effects of nerve compression on fast axonal transport in streptozotocin-induced diabetes mellitus. An experimental study in the sciatic nerve of rats. Diabetologia 29: 181–185. 2422081 10.1007/BF02427090

[29] Fink DJ, Purkiss D, Mata M (1987) Alterations in retrograde axonal transport in streptozocin-induced diabetic rats. Diabetes 36: 996–1000. 2440748 10.2337/diab.36.9.996

[30] Tesfaye S, Malik R, Harris N, Jakubowski JJ, Mody C, et al (1996) Arterio-venous shunting and proliferating new vessels in acute painful neuropathy of rapid glycaemic control (insulin neuritis). Diabetologia 39: 329–335. 8721779 10.1007/BF00418349

[31] Vinik A (2010) The approach to the management of the patient with neuropathic pain. J Clin Endocrinol Metab 95: 4802–4811. 21051576 10.1210/jc.2010-0892

[32] Tesfaye S, Kempler P (2005) Painful diabetic neuropathy. Diabetologia 48: 805–807. 15834549 10.1007/s00125-005-1721-7

[33] Ueda H (2006) Molecular mechanisms of neuropathic pain-phenotypic switch and initiation mechanisms. Pharmacol Ther 109: 57–77. 16023729 10.1016/j.pharmthera.2005.06.003

[34] DeJong RN (1977) CNS manifestations of diabetes mellitus. Postgrad Med 61: 101–107. 834675 10.1080/00325481.1977.11714510

[35] Suzuki C, Ozaki I, Tanosaki M, Suda T, Baba M, et al (2000) Peripheral and central conduction abnormalities in diabetes mellitus. Neurology 54: 1932–1937. 10822432 10.1212/wnl.54.10.1932

[36] Eaton SE, Harris ND, Rajbhandari SM, Greenwood P, Wilkinson ID, et al (2001) Spinal-cord involvement in diabetic peripheral neuropathy. Lancet 358: 35–36. 11454377 10.1016/S0140-6736(00)05268-5

[37] Kapur D (2003) Neuropathic pain and diabetes. Diabetes Metab Res Rev 19 Suppl 1S9–15. 12577253 10.1002/dmrr.359

[38] Barrett SL, Dellon AL, Fleischli J, Gould JS, Wang C (2010) Metabolic and compressive neuropathy. Foot Ankle Spec 3: 132–139. 20508014 10.1177/1938640010368028

[39] Jensen TS, Baron R (2003) Translation of symptoms and signs into mechanisms in neuropathic pain. Pain 102: 1–8. 12620591 10.1016/s0304-3959(03)00006-x

[40] Dellon AL, Muse VL, Scott ND, Akre T, Anderson SR, et al (2012) A positive Tinel sign as predictor of pain relief or sensory recovery after decompression of chronic tibial nerve compression in patients with diabetic neuropathy. J Reconstr Microsurg 28: 235–240. 22411625 10.1055/s-0032-1306371

[41] Valdivia Valdivia JM, Weinand M, Maloney CT Jr, Blount AL, Dellon AL (2013) Surgical treatment of superimposed, lower extremity, peripheral nerve entrapments with diabetic and idiopathic neuropathy. Ann Plast Surg 70: 675–679. 23673565 10.1097/SAP.0b013e3182764fb0

[42] Hollis Caffee H (2000) Treatment of diabetic neuropathy by decompression of the posterior tibial nerve. Plast Reconstr Surg 106: 813–815. 11007393 10.1097/00006534-200009020-00009

[43] Aszmann OC, Kress KM, Dellon AL (2000) Results of decompression of peripheral nerves in diabetics: a prospective, blinded study. Plast Reconstr Surg 106: 816–822. 11007394 10.1097/00006534-200009040-00010

[44] Therapeutics, Technology Assessment Subcommittee of the American Academy of N, Chaudhry V, Stevens JC, Kincaid J, et al (2006) Practice Advisory: utility of surgical decompression for treatment of diabetic neuropathy: report of the Therapeutics and Technology Assessment Subcommittee of the American Academy of Neurology. Neurology 66: 1805–1808. 16801641 10.1212/01.wnl.0000219631.89207.a9

[45] Cornblath DR, Vinik A, Feldman E, Freeman R, Boulton AJ (2007) Surgical decompression for diabetic sensorimotor polyneuropathy. Diabetes Care 30: 421–422. 17259523 10.2337/dc06-2324

[46] Chaudhry V, Russell J, Belzberg A (2008) Decompressive surgery of lower limbs for symmetrical diabetic peripheral neuropathy. Cochrane Database Syst Rev: CD006152. 10.1002/14651858.CD006152.pub2 PMC8990523 18646138

